# Strategies to promote safe mobilization of people with obesity in inpatient care settings: A scoping review protocol

**DOI:** 10.1371/journal.pone.0353176

**Published:** 2026-07-06

**Authors:** Rebecca A. Roberts, Elizabeth Finkelzon, Anika Younker, Georgia Merriman, Paul Claveria, Julianna Lancaster, Christopher Farley, Anastasia N. L. Newman

**Affiliations:** School of Rehabilitation Science, Faculty of Health Science, McMaster University, Hamilton, ON, Canada; University of Saskatchewan, CANADA

## Abstract

**Introduction:**

Hospital-associated deconditioning is common during inpatient admissions, particularly among patients with obesity whose mobilization presents added complexity and safety challenges. Despite the known benefits of mobilization, there is no comprehensive synthesis or guidelines to address safe mobilization for patients with obesity in inpatient settings.

**Materials and methods:**

This protocol describes a scoping review that will systematically map the literature on mobilization strategies used for adults with obesity in acute and inpatient rehabilitation settings, including barriers and facilitators to safe practice. Following the Joanna Briggs Institute methods and PRISMA-ScR reporting, peer-reviewed studies involving patients with author-defined obesity will be identified through comprehensive searches of Ovid MEDLINE, Ovid Embase, Ovid Emcare, Ovid AMED, EbscoHost CINAHL, and Clarivate Web of Science from database inception. Screening and data extraction will be conducted independently by two reviewers, with discrepancies resolved by consensus or a third reviewer. Data will be summarized narratively and with descriptive statistics, as appropriate.

**Conclusion:**

The results of this scoping review will summarize the available evidence on the strategies used to safely mobilize patients with obesity during admission to inpatient settings and the barriers and facilitators that impact these strategies. A manuscript will be submitted for publication in a peer-reviewed journal and presented at appropriate rehabilitation conferences.

## Introduction

Admission to acute care settings is associated with increased risk of infections, pressure ulcers, falls, and hospital-associated deconditioning [1]. Hospital-associated deconditioning refers to the reduction in cognitive and physical functioning that results from a lack of activity and can impair a person’s ability to perform activities of daily living [1]. Mobilization during an acute care admission involves engaging patients in movement; it can improve physical functioning, decrease hospital length of stay, and reduce complications, such as delirium, thromboembolic events, and psychological distress [2,3]. However, implementing mobilization within inpatient settings can be challenging with risks that include mechanical falls, unplanned removal of lines and tubes, oxygen desaturation, and hemodynamic changes [2]. For patients with obesity, mobilization requires careful consideration of additional safety factors. Safe mobilization of patients with obesity often requires additional planning, more staff, specialized bariatric equipment, and patient education [4]. Absence of these resources may pose a challenge for maintaining the safety of both the patient and the staff involved [4].

In 2021, an estimated 2.11 billion adults were classified as overweight or obese worldwide [5]. Globally, adult obesity has more than doubled since 1990, and adolescent obesity has quadrupled, placing a greater number of individuals at risk for obesity-related disease and mortality [6,7]. Obesity is a complex, chronic, multifactorial disease characterized by excessive accumulation of adipose tissue to an extent that impairs health and poses implications for healthcare delivery [8].

Within inpatient settings, obesity can pose health and safety risks to both patients and health care professionals. Evidence suggests that obesity increases the economic burden on healthcare systems due to lifting injuries among health care professionals, longer patient admissions, bariatric equipment costs, and higher medical complication rates [3,9]. This burden reflects the increased complexity of care for patients with obesity due to comorbidities, respiratory complications, and mobility challenges [3,9]. Mobilization during admission to inpatient settings is vital to reduce complications, improve functional outcomes, prevent deconditioning, and decrease length of stay, particularly when patients have obesity [10,11]. However, there are barriers to mobilizing patients with obesity, including a lack of appropriate equipment, staff discomfort or fear, inadequate staffing, and safety concerns [6,12]. Additionally, weight-based stereotypes held by some health care professionals can result in judgmental behaviors, lowered expectations for recovery, and diminished quality of care, which present barriers to effective mobilization of patients with obesity [13,14]. Although literature exists supporting both the benefits and challenges associated with early and safe mobilization, there remains limited synthesized evidence addressing mobilization strategies for patients with obesity in inpatient settings.

To facilitate safe mobilization of patients with obesity, there is a need to identify and understand the strategies or interventions that have been utilized to promote mobilization with this population. Additionally, staff perspectives, system-level factors (e.g., training, equipment availability, and institutional protocols), and barriers and facilitators that influence mobilization practices need to be further explored [8]. To our knowledge, this is the first scoping review to comprehensively map the literature on the safe mobilization of patients with obesity in inpatient care settings. Evidence specific to adults with obesity remains fragmented across different settings, interventions, and implementation contexts. This review aims to identify and synthesize existing evidence on mobilization strategies used with adults with obesity within inpatient settings, helping to clarify what approaches are being used, where the evidence is concentrated, and where gaps in the literature remain. Our secondary objective is to explore the barriers and facilitators that influence the safe mobilization of adults with obesity within these settings in order to identify modifiable factors relevant to clinical practice, education, and patient safety. In addition to describing current practice, this review will identify contextual and behavioural factors associated with implementation, with the goal of informing future practice, training, and service design. To this end, our research questions are: What strategies are used to safely mobilize adults with obesity during admission to inpatient settings, and what are the barriers and facilitators to this mobilization?

## Materials and methods

This scoping review will be conducted in accordance with the Joanna Briggs Institute (JBI) methods [15] and will be reported according to the Preferred Reporting Items for Systematic Reviews and Meta-Analyses extension for Scoping Reviews (PRISMA-ScR) (S1 Table) [16]. Our study has been registered a priori with Open Science Framework.

### Eligibility criteria

We utilized the JBI Population, Concept, Context (PCC) [15] framework to guide the development of our eligibility criteria (Table 1). Peer-reviewed full-text publications will be included if they (1) enrolled adults (aged ≥18 years old) with obesity who were admitted to an acute care or inpatient rehabilitation setting, and (2) report strategies to facilitate mobilization (e.g., physical interventions, equipment use, staff education, multidisciplinary approaches), or discuss the barriers and facilitators to safely mobilizing patients with obesity. Obesity will be defined as per the authors’ of the included publications; anticipated definitions of obesity include Body Mass Index (BMI) >30 [5], Class I-III obesity, or Edmonton Obesity Staging System (EOSS) scores >1 [17]. Where studies enroll some participants that do not meet our criteria, we will include a publication if at least 50% of participants meet our criteria. We will exclude citations published in a language other than English, French, and Portuguese, grey literature, conference abstracts, letters to the editor, review articles, and studies set in long-term care and retirement residences. We restricted inclusion to peer-reviewed full-text studies to ensure methodological consistency, rigour, and synthesis feasibility.

**Table 1:** Eligibility criteria.

Domain	Inclusion criteria
Population	Adults (≥18 years old) with author-defined obesity admitted to an acute care or inpatient rehabilitation setting.
Concept	Identification of strategies to facilitate mobilization of patients with obesity (i.e., physical interventions, equipment use, staff education, multidisciplinary approaches). Identification of barriers and facilitators to safe mobilization practices. Implementation of organizational, environmental, and/or educational interventions aimed at improving mobilization.
Context	Acute care or inpatient rehabilitation settings (e.g., intensive care units, medical/surgical wards, emergency departments, inpatient rehabilitation units).
Miscellaneous	Qualitative, quantitative, mixed-methods study designs. Peer-reviewed articles. Published languages – English, French, Portuguese.

### Information sources

Six databases will be searched from their inception: Ovid MEDLINE, Ovid Embase, Ovid Emcare, Ovid AMED, EbscoHost CINAHL, and Clarivate Web of Science. Additionally, the reference lists of eligible studies will be screened for other potentially relevant articles.

### Search strategy

The search strategy for this review will be developed in consultation with a health sciences librarian using the search concepts “obesity”, “inpatient”, and “mobilization”. Controlled vocabulary (i.e., subject headings) and keywords of their synonyms will be combined using Boolean operators, as appropriate. A preliminary search strategy is provided in Table 2, which will be adapted to each database. A validation exercise will be conducted for each database to verify that the search strategy successfully retrieves three previously identified relevant studies [10,12,18].

**Table 2:** Preliminary medline search strategy.

Concept	Search strategy
Obesity	1. exp Obesity/
	2. exp Obesity, Morbid/
	3. obes*.mp.
	4. exp Bariatric Surgery/
	5. exp Bariatrics/
	6. bariatric*.mp.
	7. Overweight/
	8. Overweight.mp.
	9. Adiposity/
	10. Adipose Tissue/
	11. adipos*.mp.
	12. Body composition/
	13. (body adj2 (composition or fat or weight)).ti,ab.
	14. Body Weight/
	15. fatness.ti,ab.
	16. 1 or 2 or 3 or 4 or 5 or 6 or 7 or 8 or 9 or 10 or 11 or 12 or 13 or 14 or 15
Inpatient	17. exp Hospitals/
	18. exp Hospitalization/
	19. hospital*.mp.
	20. intensive care units/ or burn units/ or coronary care units/ or respiratory care units/
	21. (ICU or CCU).mp.
	22. (intensive care unit* or burn unit* or coronary care unit* or respiratory care unit*).mp.
	23. Patient Admission/
	24. (admit* or admission).mp.
	25. Rehabilitation Centers/
	26. rehab* cent*.mp.
	27. Critical Care/
	28. critical care.mp.
	29. Inpatients/
	30. inpatient*.mp.
	31. acute care.mp.
	32. 17 or 18 or 19 or 20 or 21 or 22 or 23 or 24 or 27 or 28 or 29 or 30 or 31
Mobilization	33. Early Ambulation/
	34. ambulat*.mp.
	35. exp Walking/
	36. walk*.mp.
	37. mobili*.mp.
	38. Patient Transfer/
	39. (patient* adj2 transfer*).mp.
	40. “Moving and Lifting Patients”/
	41. ((moving or lifting) adj2 patient*).mp.
	42. mechanical lift.mp.
	43. (bed to chair or chair to bed or bed to bed).mp.
	44. Patient Positioning/
	45. patient* position*.mp.
	46. occupational therapists/ or physical therapists/
	47. (physiotherap* or physical therapy*).mp.
	48. occupational therap*.mp.
	49. 33 or 34 or 35 or 36 or 37 or 38 or 39 or 40 or 41 or 42 or 43 or 44 or 45 or 46 or 47 or 48
Combining concepts	50. 16 and 32 and 49

### Data screening and extraction

Identified citations will be uploaded to Covidence, a web-based systematic review platform (2026, Veritas Health Innovation, Melbourne, VIC, Australia) where deduplication will be completed automatically. Title/abstract screening and full-text selection will be performed independently and in duplicate. Any citation deemed potentially eligible by at least one reviewer during title/abstract screening will be included in full text selection. Discrepancies during full text selection will be addressed through discussion, and adjudicated by a third reviewer, if required. During full text selection, the most relevant reason for exclusion will be recorded. The results of the search and the study selection process will be reported in a PRISMA flow diagram [16].

Two reviewers will independently complete data extraction, with any discrepancies resolved via discussion or a third reviewer, as needed. Data extraction will be completed using a predefined table in Microsoft Excel. We will extract the following data from each included full text: (1) study details (i.e., title, first author, year of publication, journal title, country of origin, trial registration number, study design, study objectives, and funding sources), (2) sample characteristics (i.e., sample size, age, sex, admission diagnoses, comorbidities, and BMI), (3) setting (i.e., acute care, inpatient rehabilitation), (4) type of inpatient rehabilitation program (i.e., slow stream, high intensity, geriatric, musculoskeletal), (5) mobilization strategies (i.e., physical handling or transfer techniques, bariatric or assistive equipment use, staffing or workflow modifications, multidisciplinary approaches, staff education or training, and organizational, environmental, or policy-level supports), (6) adverse events and/or staff/patient safety concerns, and (7) barriers and facilitators to safe mobilization. We expect screening to begin in late February 2026 with data extraction occurring in April 2026.

Extraction and analysis of mobilization strategies, barriers and facilitators will be guided by the COM-B Framework [19]. The COM-B will be used as an analytic framework to interpret the behavioural and contextual determinants that influence mobilization practices. The framework explains that behaviour occurs as an interaction of three components: (1) Capability (psychological and physical), (2) Opportunity (social and environmental), and (3) Motivation (conscious and automatic) [19].

### Data analysis

We will use narrative analysis and descriptive statistics to summarize and present extracted data. Descriptive statistics will be presented as counts (percentages), means (standard deviations), and medians (first and third quartiles).

## Discussion

This scoping review will comprehensively map and synthesize the current evidence on mobilization practices for individuals with obesity within inpatient settings. By examining both current strategies and the contextual factors that influence their implementation, the review will help identify modifiable barriers and facilitators that may support safer and more effective mobilization within acute care and inpatient rehabilitation settings. These findings may inform clinical practice, policies, and continuing education for healthcare professionals involved in the mobilization of individuals with obesity. They may also support more equitable access to rehabilitation and help reduce work-related injuries among clinicians, such as physiotherapists. Findings will be disseminated through publication and presentation at relevant rehabilitation conferences.

This scoping review will have several strengths, including adherence to JBI methodology, a comprehensive search of peer-reviewed literature across multiple databases developed in consultation with a health sciences librarian, and a structured approach to interpreting barriers and facilitators which will be grounded in the COM-B framework. This scoping review will have limitations. Due to the language expertise of the review team, we will only include peer-reviewed studies in English, French, and Portuguese. To manage the scope of the review, we have opted to exclude grey literature.

## Supporting information

S1 Table PRISMA-P Checklist. (DOCX)
